# Two-stage window screening and development trajectories in early identification of autism spectrum disorder among Han Chinese children

**DOI:** 10.1186/s13104-021-05548-1

**Published:** 2021-04-07

**Authors:** For-Wey Lung, Bih-Ching Shu

**Affiliations:** 1 Calo Psychiatric Center, Pingtung County, Taiwan; 2 Graduate Institute of Medical Science, National Defense Medical Center, Taipei, Taiwan; 3 International Graduate Program of Education and Human Development, National Sun Yat-Sen University, Kaohsiung, Taiwan; 4 Institute of Education, National Sun Yat-Sen University, Kaohsiung, Taiwan; 5 Institute of Allied Health Sciences and Department of Nursing, National Cheng Kung University, No. 1 Da-Hsueh Rd., Tainan, 701 Taiwan

**Keywords:** Taiwan Birth Cohort Study, Child development trajectory, Autism spectrum disorder, DSM-5

## Abstract

**Objective:**

An understanding of the trajectory and norm of development in children is needed in order to understand the concept of the spectrum in the diagnosis of autism spectrum disorder (ASD). Children’s developmental growth trajectory was measured from six to 66 months in the Taiwan Birth Cohort Study dataset (N = 11,145). Additionally, over 4 years of follow-up, the negative predictive value of using the Parental Concern Checklist and Taiwan Birth Cohort Study Developmental Instrument was also investigated as the first stage of screening in a two-stage window screening method for ASD diagnosis.

**Results:**

The growth trajectory showed that children’s language development began to increase at 18 months, and peaked at 36 months. On the other hand, social development showed steady growth from 18 to 66 months. The increase in the trajectory of children’s language development prior to age three, when compared with other developmental dimensions, may increase the difficulty of diagnosing ASD. The two-stage window screening method can be used in settings where the screening sample is large, such as in community or primary care settings, and has been found to be time- and cost-efficient. Better understanding of children’s developmental trajectory can enhance detection and intervention for ASD.

## Introduction

With the production of the fifth edition of the *Diagnostic and Statistical Manual of Mental Disorders* (*DSM-5*), many issues have been raised that are affected by our conceptualization of each psychiatric diagnosis. To find universal criteria that can be used in all cultures is difficult; however, this issue affects each diagnosis made. With an increased prevalence of autism spectrum disorder (ASD) from 1 in 54 [1], compared with an earlier prevalence of 6.7 per 1000 [2], the diagnosis of ASD has attracted increased levels of attention and concern. Screening tools for ASD have also been developed to promote early identification and intervention [3]. However, the median age of diagnosis is still past the age of 3 years [4], and even later for children from disadvantaged backgrounds [5]. For earlier detection and diagnosis, the American Academy of Pediatrics guidelines recommend routine ASD-specific and standardized developmental screening surveillance of every child. Therefore, ASD screening tools that can be used in primary care and community settings are vital.

In the community, instruments that can be completed without professional training and that are short and easily comprehensible are needed. Community screening instruments are not only helpful to the public in heightening understanding of their children’s developmental condition, they may also aid doctors in their decision-making process. Furthermore, for clinicians to interpret positive predictive values (PPV) and negative predictive values (NPV) appropriately, they have to know the estimated prevalence of the condition in their particular population [7]. Hence, to understand the idea of a spectrum in the ASD diagnosis in the *DSM-5*, we need to understand not only the etiology of ASD, but also the trajectory and norm of children’s development.

For early detection and large-scale screening in the community, two-stage screening methods are often used. A two-stage screening process involves the use of a more inexpensive, less invasive test at the first stage (e.g., Modified Checklist of Autism in Toddlers [M-CHAT]), and a more expensive or more invasive, but more sensitive and specific test at the second stage [e.g., Autism Diagnostic Inventory-Revised (ADI-R)] [6]. Chien, Huang and Lung proposed the two-stage window screening method, which is more efficient than other methods [7]. This screening method can be used in settings where the screening sample is large, such as in community or primary care settings [7]. Two cutoff points are needed in the first stage of the two-stage window screening, yielding three groups. Those in the first (highest scoring) group are normal (assuming higher scores are better), and those in the third (lowest) group have the highest risk for the disorder; therefore, they can be scheduled directly for diagnostic assessment. Participants in the middle group, between the two cutoff points, have the highest probability of being misclassified and need to be screened further using a more specific screening instrument. Fewer people therefore need to be screened in the second stage using this method, which reduces the cost of the testing [6, 7]. Lung and colleagues found that the Parental Concern Checklist (PCC) and Taiwan Birth Cohort Study Developmental Instrument (TBCS-DI) are efficient first-stage broadband screening instruments for ASD in community-based settings [8, 9].

The Taiwan Birth Cohort Study (TBCS) is a national birth cohort study dataset, designed to establish national norms of children’s development. To understand the idea of the spectrum in the diagnosis of ASD, this study used the TBCS dataset to investigate the developmental growth trajectory and norm of children’s development from 6 to 66 months in gross motor, fine motor, language, and social communication developmental dimensions. Additionally, over 4 years of follow-up, the NPV, PPV, sensitivity and specificity of using the Parental Concern Checklist (PCC) and Taiwan Birth Cohort Study Developmental Instrument (TBCS-DI) as the first-stage screening in a two-stage window screening method for ASD diagnosis were also investigated.

## Main text

### Methods

The TBCS aimed to develop a nationally representative cohort database using a national household probability sampling method. All babies born in 2005 in Taiwan were eligible for the TBCS, with no exclusion criteria. Two-stage stratified random sampling was used, with cities and towns as the primary sampling unit at the first stage, and newborns were selected proportionally according to the rate of births at the second stage. A total of 21,248 families (11.7% selection rate) participated when the children were six months old; 20,172 families agreed to be followed up at 18 months; 19,910 at 36 months; 19,721 at 66 months; and finally, 19,516 families were followed up when the children were 8 years old [10]. The procedures of the study were approved by a teaching hospital in Taiwan. Children who participated in all five stages of the study were included in the investigation of children’s developmental trajectories, resulting in a dataset of 11,145 children.

#### Materials

The PCC is eight items screening instrument which can be used as the first-stage screening instrument in a two-stage window screening method using cutoff points of 2/3 and 6/7 [8].

The TBCS-DI is a short, culturally sensitive parental-report developmental instrument measuring the children’s daily performance, which has shown good reliability and validity [11–15]. Using the TBCS-DI as the first-stage screening instrument and the M-CHAT as the lead criteria for screening for ASD at six, 18, and 36-months in a two-stage window screening method, with cutoff points of 65/66, 42/43, and 51/52 in the 6-, 18-, and 36-month scales, the TBCS-DI resulted in good NPV [9].

#### Statistical analysis

Children’s six, 18, 36, and 66-months developmental trajectory was investigated using latent growth model (LGM). LGM can investigate inter-individual differences in intra-individual changes over time, showing the initial status and trajectory of the development of each child studied. The LGM was processed using the Analysis of a MOment Structures 7.0 statistical software package (SPSS Inc., Chicago, USA).

### Results

**Fig. 1:**
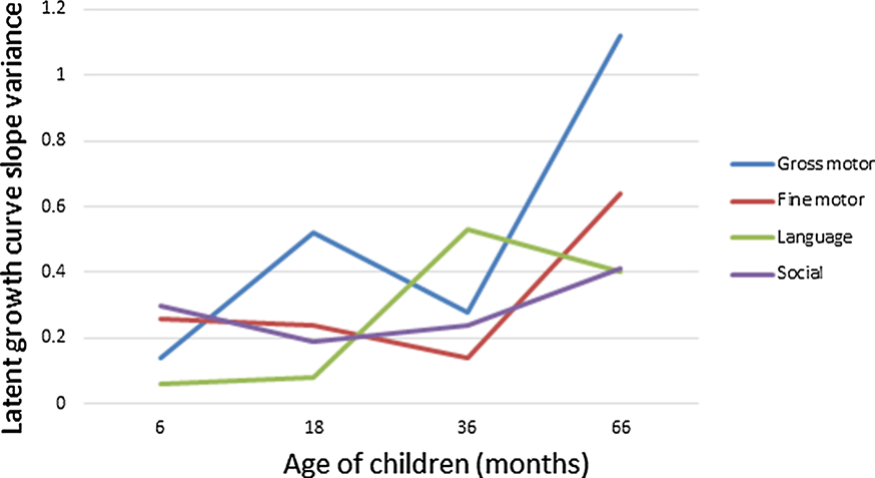
Latent growth curve of children’s developmental trajectory from 6 to 66 months in the gross motor, fine motor, language, and social communication dimensions

The LGM of the gross motor, fine motor, language, and social dimensions resulted in a good fit, with adjusted goodness-of-fit index root mean square error of approximation (RMSEA) > 0.9, and RMSEA < 0.08 (Gross motor: AGFI = 0.998, RMSEA = 0.016; fine motor: AGFI = 1.000, RMSEA < 0.001; language: AGFI = 0.982, RMSEA = 0.060; social: AGFI = 0.975, RMSEA = 0.071). Factor variance in children’s six to 66-months development in the four developmental dimensions is illustrated in Fig. 1. The LGM gross motor development variance at 6-, 18-, 36-, and 66-months resulted in 0.14, 0.52, 0.28, and 1.12; Fine motor development resulted in 0.26, 0.24, 0.14, and 0.64; Language development resulted in 0.06, 0.08, 0.53, and 0.40; and social development in 0.30, 0.19, 0.24, and 0.41.

The NPV, PPV, sensitivity and specificity of the PCC for screening children at 18 months, and their rate of receiving a diagnosis of ASD when the children were 66 months old is investigated. The results showed that, after 4 years, the PPV, NPV, sensitivity and specificity of the PCC resulted in 0.95%, 99.67%, 24.41%, and 87.87%, respectively, as shown in Table 1.

**Table 1:** The application of the two-stage window screening method for screening of children using the Parental Concern Checklist (PCC) at 18 months and the rate of diagnosis with autism spectrum disorder (ASD) at 5.5 years old

	ASD	Not diagnosed	Total	
PCC 3–6	25	2615	2640	PPV: 25/2640 = 0.95%
Others	63	18,960	19,023	NPV: 18,960/19,023 = 99.67%
Total	88	21,575	21,663	
	Sensitivity 25/88 = 28.41%	Specificity 18,960/21,575 = 87.87%		

*PPV* positive predictive value, *NPV* negative predictive value

Using the TBCS-DI as the first-stage screening instrument and the M-CHAT as the lead criteria for screening for ASD in a two-stage window screening method. The PPV, NPV, sensitivity and specificity of the TBCS-DI, with regard to the percentage of children diagnosed with ASD when they were 66 months old resulted in 1.41%, 99.81%, 61.36%, and 82.60%, respectively, as shown in Table 2.

**Table 2:** The application of the two-stage window screening method for screening of children using the Taiwan Birth Cohort Study Developmental Instrument (TBCS-DI) at 18 months and the rate of diagnosis with autism spectrum disorder (ASD) at 5.5 years old

	ASD	Not diagnosed	Total	
TBCS-DI	54	3763	3817	PPV: 54/3817 = 1.41%
Others	34	17,812	17,846	NPV: 17,812/17,846 = 99.81%
Total	88	21,575	21,663	
	Sensitivity 54/88 = 61.36%	Specificity 17,822/21,575 = 82.60%		

*PPV* positive predictive value, *NPV* negative predictive value

### Discussion

The TBCS dataset with a large sample follow-up of children’s development from six to 66-months shows that the growth trajectory of children’s language development began to increase at 18 months, and peaked at 36 months. On the other hand, children’s social development showed a steady growth from 18 to 66 months. Using the PCC and TBCS-DI as the first stage screening instrument in a two-stage window screening method for ASD, our study shows that after 4-years follow-up, the NPV of the PCC remains high at 99.67%, and TBCS-DI at 99.81%, showing that those that were screened out using these instruments were not likely to be diagnosed. Therefore, the PCC and TBCS-DI can both be used as a community screening instrument for ASD using a two-stage window screening method.

The growth trajectory of children’s development in this study is congruent with previous studies that showed that at an early age, when children’s language development is increasing, the pathological language patterns of children with ASD are categorized into the stereotyped dimension of behavior. As children grow older, and when compared with the social development of normally developing children, their language dysfunction is reflected in the category of social communication [16, 17]. The increase in the trajectory of children’s language development prior to 3 years old, when compared with other developmental dimensions, may also increase the difficulty of diagnosis of ASD.

Owing to the Chinese cultural context of collectivism, greater restraint is exerted over emotional displays than in more individualistic cultures [18]. Although the emotion dimension could not be distinguished prior to the age of eight, a predictive validity model showed it to be a suppressor within the social dimension in earlier scales [10], supporting the importance of social communication in Asian collectivist culture [18]. Differences in the emotional development of children raised in Chinese culture may also influence the ability to diagnose atypical empathic responses in children with ASD [19].

Using the PCC and TBCS-DI as the first-stage screening instrument in a two-stage window screening method, the results showed that, after 4 years, the NPV of the PCC and TBCS-DI remained high, showing that those who were not screened out at 18 months were unlikely to be diagnosed 4 years later with ASD. In the traditional method of positive or negative two-stage screening, all those scoring higher than the cutoff point need testing at the second stage. Fewer people need to be assessed in the second stage using the two-stage window screening, which decreases the cost of testing [7] and the number of false positives [8]. Although both TBCS-DI and PCC are parental reports of their children’s development, however, while the PCC deals with parental concerns, the TBCS-DI is focused on children’s developmental milestones. In clinical research, the prevalence is set at 50% (control vs. clinical groups) and is not reflective of the real prevalence of the disorder. Therefore, low PPVs are accepted in community research because the threshold for failing the screening was set low to prevent as many missed diagnoses as possible at the expense of the PPVs. Therefore, even though low PPVs may bring about a high rate of false positives, causing over-referrals in the community, a study showed that those who screen out as false positives in developmental screening perform substantially worse than the true negatives in standardized testing, and show greater psychosocial risks [20]. For that reason, early intervention for those screened out as false positives, as a preventive strategy, is still needed when it is permitted, because it can reduce stigmatization later on.

### Conclusion

With the launch of the *DSM-5*, studies have pointed to the greater validity of the new autism dyad [16, 17], with the core impairments of ASD being the expression of separable social communication and restricted and repetitive behavior and interest dimensions. This has decreased false positive diagnoses and simultaneously maintained or improved identification of ASD cases [21]. Our national birth cohort follow-up developmental trajectory from six to 66 months of age also shows language development to be unstable prior to 36 months. Social development is more homogeneous, showing a steady growth from 18 to 66 months, thus it is a more reliable factor for diagnosis. Enhancement of ASD detection can provide earlier and more appropriate intervention. In order to appropriately for and identify children with ASD from diverse backgrounds, we need to understand the normal trajectory of development across different sectors of society. Cultural differences in normative social and emotional development may contribute to cultural differences in ASD symptom presentation, and to disparities in outcomes for children with ASD from diverse backgrounds. Future follow-up of the TBCS will hopefully provide us with more information regarding the application of the *DSM-5* and the etiology of factors that may affect the development of children in the Chinese community.

## Limitation

A limitation of our study is that the clinical diagnosis of ASD was based on parental reporting. However, nationally representative parental surveys have shown ASD prevalence compatible with estimates from population-based studies that relied on medical and special education records [22].

## Data Availability

Taiwan Birth Cohort Study datasets can be obtained from the Taiwan Ministry of Health and Welfare, Bureau of Health Promotion, Taiwan. https://dep.mohw.gov.tw/DOS/np-2500-113.html

## Abbreviations

ADI-R: Autism Diagnostic Inventory-Revised
ASD: Autism spectrum disorder
DSM-5: Diagnostic and Statistical Manual of Mental Disorders
M-CHAT: Modified Checklist of Autism in Toddlers
NPV: Negative predictive value
TBCS: Taiwan Birth Cohort Study
TBCS-DI: Taiwan Birth Cohort Study Developmental Instrument
